# Living kidney transplantation between brothers with unrecognized renal amyloidosis as the first manifestation of familial Mediterranean fever: a case report

**DOI:** 10.1186/s12881-017-0457-9

**Published:** 2017-08-31

**Authors:** Ramón Peces, Sara Afonso, Carlos Peces, Julián Nevado, Rafael Selgas

**Affiliations:** 1 Nephrology Department, La Paz University Hospital, IdiPAZ, Autonomous University, Madrid, Spain; 2 Information Technology Area, SESCAM, Toledo, Spain; 3 Medical and Molecular Genetics Institute (INGEMM), Basic Research Center in the Rare Diseases Network (CIBERER), La Paz University Hospital, IdiPAZ, Madrid, Spain; 4 Servicio de Nefrología, Hospital Universitario La Paz, Paseo de la Castellana 261, 28046 Madrid, Spain

**Keywords:** Amyloidosis, Anakinra, Colchicine, Familial Mediterranean fever, Living kidney transplantation, *MEFV* gene

## Abstract

**Background:**

Familial Mediterranean fever is an autosomal recessive disease characterized by recurrent episodes of fever and polyserositis and by the onset of reactive amyloid-associated amyloidosis. Amyloidosis due to familial Mediterranean fever can lead to end-stage renal disease, culminating in kidney transplantation for some patients. In this study, we report the clinical outcome of two brothers with familial Mediterranean fever who were the inadvertent donor and recipient, respectively, of a kidney. Subsequently, they were diagnosed with renal amyloidosis secondary to familial Mediterranean fever and were successfully treated with anakinra and colchicine.

**Case presentation:**

Two brothers with familial Mediterranean fever and renal amyloidosis were the inadvertent donor and recipient, respectively, of a kidney. The recipient had presented recurrent acute febrile episodes of familial Mediterranean fever, developed nephrotic syndrome secondary to amyloidosis and needed bilateral nephrectomy and chronic dialysis. His elder brother, in apparent good health, donated his left kidney to his brother. Immediately after the kidney transplantation, both the donor and recipient presented massive proteinuria, impaired renal function and elevated serum amyloid A levels. Biopsies of the brothers’ kidneys showed amyloidosis. Genetic studies thereafter revealed a homozygous variant for the *MEFV* gene (NM_000243.2.c.2082G > A; p.M694I) in both brothers. At this point, both the donor and recipient were treated with colchicine and anakinra, resulting in improved renal function, decreased proteinuria, undetectable serum amyloid A levels and stable renal function at 62 months of follow-up and no major adverse effects.

**Conclusions:**

In familial Mediterranean fever, analyses of the *MEFV* gene should be performed in potential live kidney donors from a direct family member (either between siblings or between parents and children). In addition, genetic studies are required when consanguinity is suspected between members involved in the living transplant. Finally, anakinra could be a safe adjuvant therapy combined with colchicine for patients with familial Mediterranean fever and amyloidosis, including those with successful kidney transplantation.

## Background

There has been a progressive increase worldwide in the number of patients with end-stage renal disease (ESRD) who require kidney transplantation. As a result, many transplantation programs have been forced to recruit more cadaveric and living donors [1, 2]. In many countries, living related donors are still the main source of kidneys for transplantation due to the poor legal definition and deficient organization required for a cadaver donor work-up [1, 2] and by the fact that recipient and graft survival rates with living donors have consistently been higher than the rates achieved with cadaver donors [3]. Once a suitable living donor has been found, it is necessary to ensure that the donor has two properly functioning kidneys and that renal function is evenly divided between them. The battery of tests for the preoperative evaluation of potential live-kidney donors is currently considered adequate for detecting or suspecting any functional or anatomical renal abnormality but does not include prior genetic tests. Performing a uninephrectomy in living related donors without first conducting genetic studies could, however, accelerate the progression of pre-existing occult primary renal disease [4, 5] in both the recipient and donor.

Amyloidosis due to familial Mediterranean fever (FMF) can lead to ESRD, culminating in kidney transplantation for some patients [6]. Amyloidosis can develop in patients who have never experienced clinical symptoms of FMF (known as phenotype 2) [6, 7]. Surprisingly, there are currently no available data regarding the clinical outcomes of living relative donors who carry amyloidosis due to FMF. This report is, to our knowledge, the first case of FMF and unrecognized renal amyloidosis in both donor and recipient after living related kidney transplantation involving a pair of brothers with the same homozygous *MEFV* gene mutation (p.M694I/p.M694I). We discuss the current evidence on this topic and summarize the exceptional experience of two FMF patients with severe FMF mutations and divided renal mass, who had renal amyloidosis and were successfully treated with colchicine and anakinra.

## Case presentation

### Patient population

#### Recipient

A 31-year-old man, originally from Morocco, was admitted to Leiden University Medical Center (LUMC, The Netherlands) complaining of leg edema in February 2000. The patient had a history of recurrent episodes of inflammation, fever and serositis. The patient was diagnosed with nephrotic syndrome due to amyloidosis secondary to FMF. His family had 7 siblings with consanguineous parents. At that time, no genetic mutations were found. After another episode of FMF in 2009, the patient presented with renal failure and nephrotic syndrome that did not respond to conservative therapy (angiotensin-converting-enzyme inhibitors, diuretics, nonsteroidal anti-inflammatory drugs, cyclosporine and colchicine). He was suspected not to be compliant with treatment. A bilateral nephrectomy was performed to halt the progressive catabolism due to unrestrained renal protein loss. The patient, a Dutch resident, required chronic dialysis. At this time, there was no evidence of clinical cardiac involvement by amyloidosis. In 2011, he received a left kidney transplant from his elder brother, a resident of Spain, who had no medical history of note (the preoperative evaluation of his clinical status and renal function were unremarkable). The kidney donation and transplantation were performed in the Netherlands. The recipient received a standard immunosuppression protocol (cyclosporine, mycophenolic acid and prednisolone). After transplantation, the recipient’s postoperative course was unsatisfactory, with a 10% decrease in serum creatinine levels on 3 consecutive days during the first postoperative week. His renal function subsequently improved, although slowly, and he developed massive proteinuria (18 g/day) and elevated serum amyloid A levels and was diagnosed with nephrotic syndrome. A graft transplant biopsy showed extensive amyloid deposition. The patient underwent treatment with colchicine (0.5 mg/12 h, adjusted for renal function), anakinra (100 mg/day), prednisolone (10 mg/day), mycophenolic acid (540 mg/12 h) and cyclosporine (150 mg/12 h). After 6 months, the patient presented a serum creatinine level of 2.10 mg/dL (185.6 μmol/L) and proteinuria of 0.25 g/day. After 20 months, the graft was functioning well, with a serum creatinine level of 2.40 mg/dL (212.1 μmol/L), undetectable serum amyloid A levels and proteinuria of 0.20 g/day. After 62 months and at the time of this report, his serum creatinine was 2.05 mg/dL (182 μmol/L), Modification of Diet in Renal Disease (MDRD) value of 35 mL/min/1.73 m^2^ and proteinuria of 0.44 g/day.

### Donor

An apparently healthy 45-year-old man, originally from Morocco and a resident of Spain for 12 years, was considered a living kidney donor for his younger brother. His medical history and examination was compatible for a living related kidney donation procedure. A thorough physical examination showed no abnormalities, and the laboratory work-up included urine analysis, urine culture, blood test and liver function, the results of which were all within normal ranges. Other studies revealed a serum creatinine level of 1 mg/dL (88.4 μmol/L), creatinine clearance of 113 mL/min/1.73 m^2^, a 24-h urine protein level of 100 mg and a C-reactive protein (CRP) level of 6 mg/dL (normal range < 5 mg/dL). A computed tomography (CT) angiography of his abdomen showed both kidneys had a perfect configuration with a single renal artery and vein, a normal excretory system and normal appearance of the bladder. After the kidney donation in 2011, the patient presented nausea, vomiting and diarrhea and developed an erysipelas-like erythema of the lower legs. On the fifth postoperative day, he presented a serum creatinine level of 2.08 mg/dL (184 μmol/L). With the diagnosis of prerenal renal failure, the patient was started on IV fluids, which resulted in partially improved renal function. He was released from hospital on the seventh day after the kidney transplantation and was readmitted 3 days later for dyspnea and chest pain. At that time, his serum creatinine level had increased to 2.20 mg/dL (197 μmol/L). Nineteen days after the transplantation, the patient presented a serum creatinine level of 4.80 mg/dL (424.3 μmol/L), proteinuria of 25 g/day and a serum amyloid A level of 85 mg/L. A renal biopsy showed 8 glomeruli, one of which was globally sclerosed and all were pathological. There was extensive expansion of the mesangium, with periodic acid-Schiff (PAS)-positive eosinophilic material, which also caused occlusion of the glomerular capillaries. The eosinophilic material also invaded the blood vessels and was stained with Congo Red. The interstitium presented fibrosis and a diffuse lymphocytic infiltrate. There was also a component of acute tubular necrosis with regeneration. The patient underwent treatment with colchicine (0.5-1 mg/12 h, adjusted for renal function) and anakinra (100 mg/day). After 4 weeks, the patient presented a serum creatinine level of 3.80 mg/dL (339 μmol/L), proteinuria of 16 g/day and an undetectable serum amyloid A level. Treatment with candesartan (2 mg/day) was then started. Fourteen weeks after the kidney donation, the patient had a serum creatinine level of 1.93 mg/dL (171 μmol/L) and proteinuria of 7.5 g/day. He remained on regular colchicine (0.5 mg/12 h), anakinra (100 mg/48 h), candesartan (4 mg/day) and enalapril (5 mg/day) for 4 months. After 20 months, the patient was in good clinical condition. The serum creatinine level had stabilized at 1.50 mg/dL (132.6 μmol/L), creatinine clearance was 61 mL/min/1.73 m^2^ (MDRD of 53.3 mL/min/1.73 m^2^), and proteinuria was 2.6 g/day. An echocardiogram revealed an ejection fraction of 60%. Although the possibility of anakinra withdrawal was considered, we decided on indefinite therapy with anakinra and colchicine due to the satisfactory response. After 62 months and at the time of this report, the patient was taking colchicine (0.5 mg/12 h), anakinra (100 mg/48 h) and atorvastatin (40 mg/day). His serum creatinine level was 1.40 mg/dL (123.7 μmol/L), his creatinine clearance was 67.4 mL/min/1.73 m^2^ (MDRD of 55.7 mL/min/1.73 m^2^) and his proteinuria was 1.1 g/day.

### Molecular analysis

An initial genetic study on the recipient was conducted in 2000 with negative results. A second genetic analysis was performed in 2011 after the living transplantation on both brothers. Briefly, genomic DNA was prepared from 200 μL of whole blood, using a commercial kit. Hot spot testing for exons 2 (c.278 to c.910) and exon 10 (c.1900 to c.2346) of the FMF gene (*MEFV*), as well as exons 3, 4-5, 6-7 including introns 2,4 and exons 6 9, 11, by direct PCR and automated sequencing revealed homozygosity in the *MEFV* gene (c.2082G > A; p.M694I; National Amyloidosis Centre, University College London Medical School, London, UK). The reason p.M694I was not found in 2000 is not known, given that this founder mutation had been already described in 1998 [8, 9]. This missense mutation has a founder effect in the African Mediterranean population (Lebanon 12.6%; Tunisia; 13%, Syria 4.8%; Egypt 20.6%), and dates back more than 8500 years (Lebanon) (Mediterranean Founder Mutation Database). This change has been established as a pathogenic variant (Clin Var: RCV000220431.2) in a recessive condition.

## Discussion and Conclusions

FMF is caused by mutations in the *MEFV* gene located on chromosome 16p13.3, which is composed of 10 exons and encodes the 781-amino-acid protein known as pyrin/marenostrin, which regulates inflammation by modulating the IL-1 pathway [7, 10]. To date, more than 150 gene alterations (mutations/polymorphisms) located in the *MEFV* gene have been identified [7, 11]. The hotspot changes p.M694 V, p.V726A, p.M694I and p.M680I in exon 10 and p.E148Q in exon 2 are the most frequent among all populations and are associated with the most severe clinical outcomes, with amyloidosis occurring in 50-60% of untreated patients [11]. Serum amyloid A, the amyloidogenic precursor protein, is synthesized by the liver in response to proinflammatory cytokines (IL-1, IL-6 and tumor necrosis factor) and is then transported in plasma as a component of high-density lipoprotein [10]. Colchicine has reduced the incidence of this complication, which now only appears in untreated, undertreated and resistant patients, but colchicine is usually ineffective in patients with advanced amyloidosis. Clinically, FMF presents as 1 of 3 phenotypes: phenotype 1, which is commonly associated with recurrent short episodes of inflammation; phenotype 2, characterized by the presence of reactive amyloid-associated amyloidosis, the most severe complication of FMF, as the first clinical manifestation of the disease in an otherwise asymptomatic individual; phenotype 3, referred to as the silent homozygous or compound heterozygote state, in which 2 *MEFV* mutations are detected without signs or symptoms of FMF or of amyloidosis [7, 12, 13]. In this report, the recipient had phenotype 1 while the donor had phenotype 2 (pre-existing occult primary renal disease). After the living donation procedure, the donor developed a typical episode of FMF, which was likely induced by surgical stress. It is also plausible that nephronic reduction and concomitant hyperfiltration related to kidney donation constituted the trigger in our cases. Thus, the donor’s uninephrectomy caused a release of amyloid with an increase in the serum amyloid A level. A renal biopsy revealed extensive amyloidosis in both kidneys. Moreover, the reduction (at least) to half in terms of renal mass induced an increase in the glomerular filtration rate of the remaining nephrons, leading to glomerular hypertension and proteinuria in the one kidney with pre-existing amyloidosis. In this way, glomerular hypertension could exacerbate the sclerosing glomerulopathy of amyloidosis and lead to proteinuria and renal failure in both the donor and recipient. This form of presentation as a rapidly progressive amyloidosis was compatible with the termed “amyloid kidney storm”, a phenomenon seen rarely in FMF patients [14]. On the other hand, since the donor with phenotype 2 had the same mutation as the recipient, the chances were high that the donor would have developed renal failure in the foreseeable future, even if he had not donated a kidney.

Unfortunately, the genotype-phenotype correlation in FMF is not well established, and there are unexplained ethnic differences in amyloidosis rates [7, 12, 15]. These brothers who shared a common genotype (p.M694I/p.M694I) presented different phenotypic characteristics: one complained of intermittent abdominal pain, arthritis and fever and developed ESRD secondary to renal amyloidosis, while the other was asymptomatic prior to donation. The observation of different phenotypic presentations with a common genotype in two family members shows that each phenotype cannot be explained by particular mutations. This intrafamilial variability in the clinical expression could be explained by the action of modifying genes concomitant to the *MEFV* gene mutations. Another possible explanation for intrafamilial variability could be the characteristics of phenotype 2 FMF in the donor, as occurred in this case. A recent series established a frequency of 22/420 phenotype 2 FMF patients [13]. Therefore, phenotype 2 FMF is not as rare as was once thought, which should be kept in mind for all patients with unexplained proteinuria and/or acute phase response in high-risk ethnic groups for FMF. To understand the correlation between genotypic and phenotypic FMF variants, the presence of complex alleles, modifier loci, genetic heterogeneity and possible epigenetic factors should be studied extensively.

Regular prophylactic treatment with colchicine prevents or substantially reduces the clinical manifestations of FMF in at least 90% of cases. Patients with M694I therefore showed a favorable response to colchicine therapy, while those with P369S and R408Q did not [15]. Colchicine reduces the inflammatory response by preventing activation, degranulation and migration of neutrophils, binding to β-tubulin and leading to β-tubulin-colchicine complexes [16, 17]. Anakinra (an IL-1 receptor antagonist) is a novel therapeutic alternative that has been used as an adjunct therapy [16–22]. There has been no previous reports of living kidney transplantation from a donor with FMF to a recipient who also has FMF, with colchicine and anakinra treatment then initiated for both the recipient and the donor.

Both brothers were started on colchicine and anakinra to minimize further amyloid production, resulting in improved renal function and decreased proteinuria. They appeared to respond to colchicine and anakinra with complete remission of their FMF episodes and an improvement in renal function and the nephrotic syndrome. Due to the extensive amount of amyloid observed in the kidney biopsies, we decided to continue with indefinite treatment with anakinra and colchicine. Thus, for both brothers, colchicine and anakinra resulted in not only an amelioration of the acute febrile episodes of FMF inflammation but also an improvement in the kidney dysfunction that result from several years of amyloidosis. To our knowledge, this is the first instance of FMF and renal amyloidosis occurring in the transplant after a living related kidney transplantation, as well as an outcome with satisfactory renal function in both donor and recipient, for a pair of brothers sharing the same homozygous *MEFV* gene mutation. It is possible that renal transplantation prevents FMF episodes in the recipient. In this case, the protective role of immunosuppressive therapy cannot be ruled out [11]. However, a recent report showed the progression of systemic amyloidosis associated with FMF in a patient who underwent a living donor renal transplantation not having received colchicine or anakinra [23]. On the other hand, there was no evidence of a drug interaction between colchicine/anakinra and the immunosuppression administered to the kidney recipient [18, 24–27]. Moreover, when we analysed the impact of renal transplantation on the status and progression of FMF and amyloidosis, there was evidence that the long-term outcomes of transplantation was similar to that in the general transplant population and maintenance colchicine, even after decreasing its dose, effectively prevents recurrence of amyloidosis in the allograft [27].

These cases reveal that the presence of phenotype 2 FMF should be considered in cases of living kidney transplantation from direct family members such as a brother or sister. In-depth analyses of the *MEFV* gene are needed to obtain an adequate diagnosis of patients with clinical suspicion of FMF and are obligatory when consanguinity is suspected. In-depth FMF analyses of the entire *MEFV* gene should be conducted in potential live kidney donors. Finally, anakinra can be a safe adjuvant therapy when combined with colchicine for patients with FMF and amyloidosis, including those with successful kidney transplantation. However, further controlled studies to better evaluate the safety and efficacy of colchicine/anakinra in the long-term treatment of patients with FMF and amyloidosis are necessary.

## Abbreviations

ACE: Angiotensin-converting-enzyme
CRP: C-reactive protein
CT: Computed tomography
DNA: Deoxyribonucleic acid
ESRD: End-stage renal disease
FMF: Familial Mediterranean fever
IV: Intravenous
MDRD: Modification of diet in renal disease
NSAIDs: Nonsteroidal anti-inflammatory drugs
PAS: Periodic acid–Schiff
PCR: Polymerase chain reaction
